# Investigating the etiologies behind emergent mass mortalities of farmed *Liza carinata* juveniles from coastal farms at Damietta, Egypt

**DOI:** 10.1038/s41598-022-19649-9

**Published:** 2022-09-27

**Authors:** Alaa Eldin Eissa, Marwa M. Attia, Mohamed Abdelsalam, Mamdouh Y. Elgendy, Mahmoud Abou-Okada, Gehad A. Ismail, Nehal A. Younis

**Affiliations:** 1 Department of Aquatic Animal Medicine and Management, Faculty of Veterinary Medicine, Cairo University, Giza, 12211 Egypt; 2 Department of Parasitology, Faculty of Veterinary Medicine, Cairo University, Giza, 12211 Egypt; 3 Department of Hydrobiology, Veterinary Research Institute, National Research Centre, Dokki, Giza, 12622 Egypt; 4 Fish Diseases Research Department, Animal Health Research Institute, Agricultural Research Center, Giza, Egypt

**Keywords:** Molecular biology, Zoology, Environmental sciences, Hydrology, Diseases, Risk factors, Signs and symptoms

## Abstract

This study aimed to identify the mortality present in private fish farm *Amyloodinium ocellatum* and *Cryptocaryon irritans* were isolated from this outbreak affecting *Liza carinata* fingerlings at an earthen-based aquaculture facility in Damietta*,* Egypt. A total of 140 moribunds, *L. carinata,* were collected from the fish ponds during the mortality events. Physico-chemical analysis of water was analyzed. The skin, fins, gills, and eyes of each fish specimen were scraped gently onto slides in areas over 2 cm area. All smears were examined separately under the light microscope. Molecular identification of the parasites using analysis of ITS rDNA regions flanking both 18S and 28S rDNA genes of *Amyloodinium* protozoa and *C. irritans*. Identities of the detected parasites were confirmed by gene sequence and phylogenetic analysis. The majority of the examined fish (90%) were infected, 66.42% had a mixed infection, and 23.57% had a single infection either with *A. ocellatum* (10.71%) or *C. irritans* (12.85%).The mean intensity of *A. ocellatum* was 16.5 ± 2.03 in the skin and 13.18 ± 1.90 in the gills of infected fish, while that of *C. irritans* was 4.75 ± 1.05 in gills and 7.43 ± 1.45 in the skin, respectively. To control the emergent mortalities, affected ponds were treated using copper sulfate pentahydrate, hydrogen peroxides solutions, and amprolium hydrochloride powder in feed. Fish across the treated ponds were gradually improved with low morbidity and mortalityrates during the treatment period. The clinical disease was almost diminished at the end of the second week of treatment. Coinciding with the clinical improvement of the treated juveniles, microscopical examination of skin/gill scraps exhibited a marked decline in the number of protozoan parasites at the end of the second week of treatment.

## Introduction

Marine aquaculture is a major economic industry in many countries. Egyptian mariculture sector is still in its early stages, and it is not fully developed as the freshwater aquaculture industry^1^. The need to expand the mariculture industry increases in Egypt due to the scarcity and limitation of freshwater resources. The enormous aquatic marine resources available in the country are anticipated to aid the prospected expansion of this sector^2^. Mariculture is mostly practiced in northern Egypt, especially in Damietta, Port Said, Alexandria, and the Suez Canal region^2^. Mullet, European sea bass, giltheadsea bream, and meager are the main cultured species^3,4^.

The family Mugilidae comprises 17 genera and around 72 species and is widely distributed worldwide. Mullets can withstand a wide salinity gradient and thrive in various environments, including marine, brackish, and even freshwater^5^. Egypt is a leading country in mullet culture. In the last 10 years, the Egyptian mullet aquaculture sector has expanded from approximately 130,000 T in 2012 to 242,061 MT in 2018^3^. Egyptian hieroglyphics depict locals fishing for mullets over a thousand years ago (2340 B.C.)^6^. The availability of wild fry sources and extensive water resources (both brackish and marine) has encouraged the rapid growth of the mullet aquaculture industry in Egypt. Six different grey mullet species are commonly cultured in Egypt; flathead grey mullet, *Mugil cephalus*, thick lip grey mullet, *Chelon labrosus*, golden grey mullet, *Liza aurata*, black keeled mullet, *Liza carinata* (*L. carinata*), thin lip mullet *L. ramada,* and leaping mullet, *Liza saliens*. Farming of Mullet in Egypt still relies on collecting wild seed since induced spawning is only done on a small scale^6^. *L. carinata* (Valenciennes, 1836), sehlia, is known to inhabit the east coast of the Mediterranean, arriving through theSuez Canal from the Red Sea, its original distribution^7^. *L. carinata* is smaller and has a slower growth rate than other mullet species, yet there is a high market demand in Egypt, leading to high prices^8,9^.

Protozoan parasites are renowned threats to mariculture operations, causing massive financial losses that necessitate effective control measures. They have the potential to devastate fisheries and have a significant impact on fish production. *Amyloodinium ocellatum (A. ocellatum*) and *Cryptocaryon irritans (C. irritans*) are two serious protozoan parasites that cause severe mortality in wild and cultured fish^10^. *A. ocellatum* is an obligate ectoparasitic marine dinoflagellate that parasitizes a broad range of marine and brackish water fishes, causinghigh mortalities.The parasite causes the skin of affected fish to become powdery or velvety, and the resultant illness is known as velvet disease or amyloodiniosis^11,12^. It mostly infects the gills, skin, fins, eyes, and buccal cavity of host fish^13^.

The life cycle of *A. ocellatum* is divided into three stages, trophont, tomont, and a flagellate dinospore, infective stage^14,15^ *A. ocellatum* attaches itself to the epithelial tissues of the fish host through rhizoids, inflicting severe physical damage to the cells, culminating in hyperplasia, inflammation, bleeding, and necrosis^11^. Significant fish mortalities occur due to osmoregulatory imbalance and subsequent bacterial infections resulting from parasite feeding activity and the detachment of large numbers of trophonts^16^. Parasite feeding activities and the detachment of large numbers of trophonts cause severe osmo-regulatory imbalance and secondary bacterial infections collectively, resulting in substantial fish death^11,16^.

*Cryptocaryon irritans* parasites are obligate ectoparasitic protozoan that infects almost all marine teleosts^17^. *C. irritans* infects a broad range of wild and farmed fish species, causing cryptocaryoniasis or marine white spot disease with substantial losses, particularly in hatchery and nursery stages^18^. *C. irritans* multiplies rapidly and invades the integument of its host, significantly impeding skin and gill functioning^19,20^. The proliferation of epidermal cells induced by parasite feeding activities is evident macroscopically as white spots. Clinical signs of cryptocaryonosis in marine fish include pinhead-sized white nodules on the skin, fins, and gills. Fish also suffer from respiratory discomfort, pale gills, and excessive mucus production^11^. *Cryptocaryon irritans* parasites have a four-stage life cycle: theront, trophont, protomont, and a final tomont phase^21^. *C. irritans* produce lymphocytic infiltration, necrosis, and varying degrees of epithelial proliferation in fish's gills and skin, similar to *Ichthyophthirius multifiliis*^21^*.*

The control of parasitic fish diseases including, *A. ocellatum* and *C. irritans* in aquaculture, is complicated by the current limited availability of efficacious licensed products and the development of antiparasitic drug resistance. Therefore, the need to develop novel, safe, effective antiparasitic drugs is increasing. Copper sulfate, acriflavine, and formalin are commonly used to treat different fish parasitic infections, but these chemicals are highly toxic to fish^22^. Application of antiparasitic treatments in fish farming facilities requires awareness of aquaculture sustainability and environmental protection.

The present study aimed to investigate infections with protozoan parasites (*A. ocellatum* and *C. irritans*) in earthen ponds reared keeled mullet, *L. carinata,* during the early summer seasonof July 2020, using both morphological and molecular techniques. Further, the study aimed to evaluate the application of a combination of copper sulfate pentahydrate, hydrogen peroxides, Glutaraldehyde/QACs combination, and amprolium to control the mixed infections of *A. ocellatum* and *C. irritans*in a clinical field trial.

## Materials and methods

### Case history and fish sampling

In July 2020, *L. carinata,* keeled mullet, fingerlings reared in earthen ponds within a private farm at Shata, Damietta, Egypt, suffered from respiratory distress, and high mortalities were investigated. *L. carinata* was stocked with a density of 10,000 fish/acre.The mean weight of *L. carinata* at the onset of mortalities was 10 ± 2 g. the daily water replenishment rate was 20% of the total volume of the pond. The farm water has a reddish-brown colour. The feeding rate was 3% of the total fish biomass delivered three times during the day. The rice bran was the main feed delivered to fingerlings of *L. carinata*. The farm uses poultry and livestock manure. No Paddlewheels aerators exist on the farm. Fish fingerlings showed signs of respiratory distress, flashing, surfacing, accumulation at water inlet, and sudden death. *L. carinata* fingerlings were seen close to the margins of the ponds with a lack of escape reflex. Several dead fish were scattered through the pond's water and banks.

A total of 140 moribunds, *L. carinata,* with mean weight, 10 ± 2 g, were collected from the fishponds during the mortality events. Wet mounts were prepared and inspected on the spot at the farm. Scrapings was obtained from the gills and skin of moribund fish specimens. The fish were transferred to the Aquatic Animal Medicine and Management Laboratory, Faculty of Veterinary Medicine, Cairo University Egypt, in isothermal boxes with ice for further analysis.

### Physico-chemical analysis of water

Dissolved oxygen (DO), temperature, pH, and turbidity, were measured in-situ in the selected fish ponds using a Multi-probe HQ40D meter (HACH LDO; PHC301 & CDC41, Germany). Salinity was recorded using a portable refractometer (ATAGO CO., LTD. Japan).Water samples were also collected and further analyzed for un-ionized ammonia (NH3), nitrite, and nitrate. Phytoplankton was collected, filtered through a 25 μm mesh, concentrated in 20 ml sterile seawater, and fixed with Lugol. Cells were counted using a Sedgwick Rafter S50 cell counter, and micro-phytoplankton counts were expressed as cells/ml according to methods described by^23^.

### Parasitological examination

The skin, fins, gills, and eyes of each fish specimen were scraped gently onto slides in areas over 2 cm area. All smears were examined separately under the light microscope, X4 to X100, using an Olympus CX41 microscope, Japan, following the clinical procedures used by Noga^11^. Morphometric analysis of the parasitic protozoan depends on fifty parasites. All measurements are in micrometers in diameters and are given as mean S.D.^24^. Prevalence and mean intensity of protozoan infestations were calculated and recorded.

### Molecular identification

The collected protozoan parasites; from the gills and skin of moribund fish; were washed several times with distilled water to remove tissue debris and mucus and then centrifuged at 2000 × *g* for 15 min. The pooled protozoan was transferred to sterilized Eppendorf tubes and preserved at − 20 °C for further molecular identification. DNA was extracted from preserved protozoan using QIAamp a DNA Mini kit (QIAGEN, Hilden, Germany). The concentration and quality of genomic DNA were investigated using the NanoDrop™ ND-1000 Spectrophotometer (Thermo Scientific, Germany). The parasitic DNA was then preserved at − 20 °C for sequencing analysis.

A fragment of ITS rDNA regions; flanking both 18S and 28S rDNA genes of *Amyloodinium* protozoan; was amplified using the following primer pair Dino 5′UF:5′-CAACCTGGTGATCCTGCCAGT-3′ and ITS R:5′-TCCCTGTTCATTCGCCATTAC-3′ as described by Levyet al.^25^. Briefly, PCR amplifications were performed using the following conditions: initial denaturation at 94 °C for 2 min, followed by 40 cycles of (94 °C for 30 s, 60 °C for 45 s, and 72 °C for 3 min), with a final extension at72 °C of 10 min. Amplicons were purified using the QIAGEN Extraction Kit protocol (Hilden, Germany). The purified amplicons were sent directly to the Macrogen sequencing company (Macrogen, Seol, South Korea) to be sequenced using ABI 3730XL DNA sequencer in both directions.

On the other hand, the amplification of the ITS- rDNA regions flanking 18S and 28S rDNA genes of the retrieved *C. irritans* was carried out using the following primers pair, P1-FW: 5′-GTTCCCCTTGAACGAGGAATTC-3′ and NC2-RV: 5′-TTAGTTTCTTTTCCTCCGCT-3′ as described by Niu et al.^26^. The amplification was started with an initial denaturation at 94 °C for 5 min, followed by 35 cycles of (94 °C for 30 s, 53 °C for 30 s and 72 °C for 1.5 min); with a final extension at 72 °C for 10 min. The amplicon was purified and sequenced as mentioned above using the same primer pair in both directions.

The Bio Edit program assembled and edited the two retrieved sequences^27^. The assembled sequences were aligned against other ITS rDNA regions of *Amyloodinium* and *Cryptocaryon* protozoan available in the database of GenBank. Finally, the sequenced ITS rDNA regions of *Amyloodinium* and *Cryptocaryon* protozoan were deposited in the GenBank.The neighbor-joining phylogenetic tree was constructed using MEGA X, with the following parameters: maximum likelihood parameter and 1000 bootstrap replicate^28^.

### Field treatment trial

Farmed fish exhibited the same previously mentioned clinical signs in the earthen pond with a stocking density of around 8500/acre were treated with the following protocol.

The treatment strategy was divided into three main successive trials as follow.

A. Initial treatment: application of copper sulfate pentahydrate 99% at a dosage of 3 kg/acre were used as an initial disinfectant on daily basis for 7 successive days at 12 p.m.^29^, concurrently hydrogen peroxide 40% solutions were added at a dosage of 6.5 L/acre during the early mornings^30^.
B. Maintenance treatment: application of Glutaraldehyde (15%)/Quaternary ammonium compounds 25% (QACs) combination at a dosage of 200 ml/acre for 3 successive days in the late afternoon^31^. To decisively boost the treatment protocol, a systemic application of Amprolium HCl was used at a dosage of 190 g/ton feed for 3 successive days. The same treatment strategy was repeated after 2 weeks^32^.
C. Supportive treatment: at the end of the second week of treatment strategy, a supportive treatmentprotocol was adopted. Briefly, the addition of a mixture of vitamin C (1.5 kg/acre) and *Saccharomyces cerevisiae* (Brewer's yeast) (1.5 kg/acre) into the pond's water in the late afternoon as a weekly routine protocol to enhance pond aquatic biota as well as fish immune barriers.

A parasitological examination was conducted at 14 days of treatment. Fish mortalities and parasitic intensities in the skin and gills of treated fish were recorded. The intensity of protozoal infection based on mucous scrapping of skin/gills at the initiation of the treatment trial (0-day) and post two weeks treatment strategy were statistically compared by paired t-test using SPSS version^26^. A probability (*P*-value) of ≤ 0.05 was assumed for statistical significance.

### Ethics approval and consent to participate

This study was approved by the Institutional Animal Care and Use Committee, Faculty of Veterinary Medicine, Cairo University, Egypt.

### Accordance with relevant guidelines and regulations

Clinical examination, dissection, sampling, sample processing, microscopical examination, molecular typing methods and field treatment trials were carried out in accordance with relevant guidelines and regulations supported with relevant references throughout the manuscript materials and methods section.

### Compliance with ARRIVE guidelines

The current study was carried out in compliance with the ARRIVE guidelines when relevant methods applied.

## Results

### Physicochemical analysis of water samples

The mean values of the physicochemical water parameters recorded in the earthen ponds were 30 °C, 32‰, 9, 3.4 mg/L, and 650.00 (NTU) for temperature, salinity, pH, dissolved oxygen, and turbidity, respectively. The average recorded levels of un-ionized ammonia (NH_3_), nitrite (NO_2_), and nitrate (NO_3_) were 1.3 mg/L and 0.98 mg/L 2.0 mg/L, respectively. The phytoplankton biomass averaged about 1675.0 (cells/mL).

### Clinical examination

**Figure 1:**
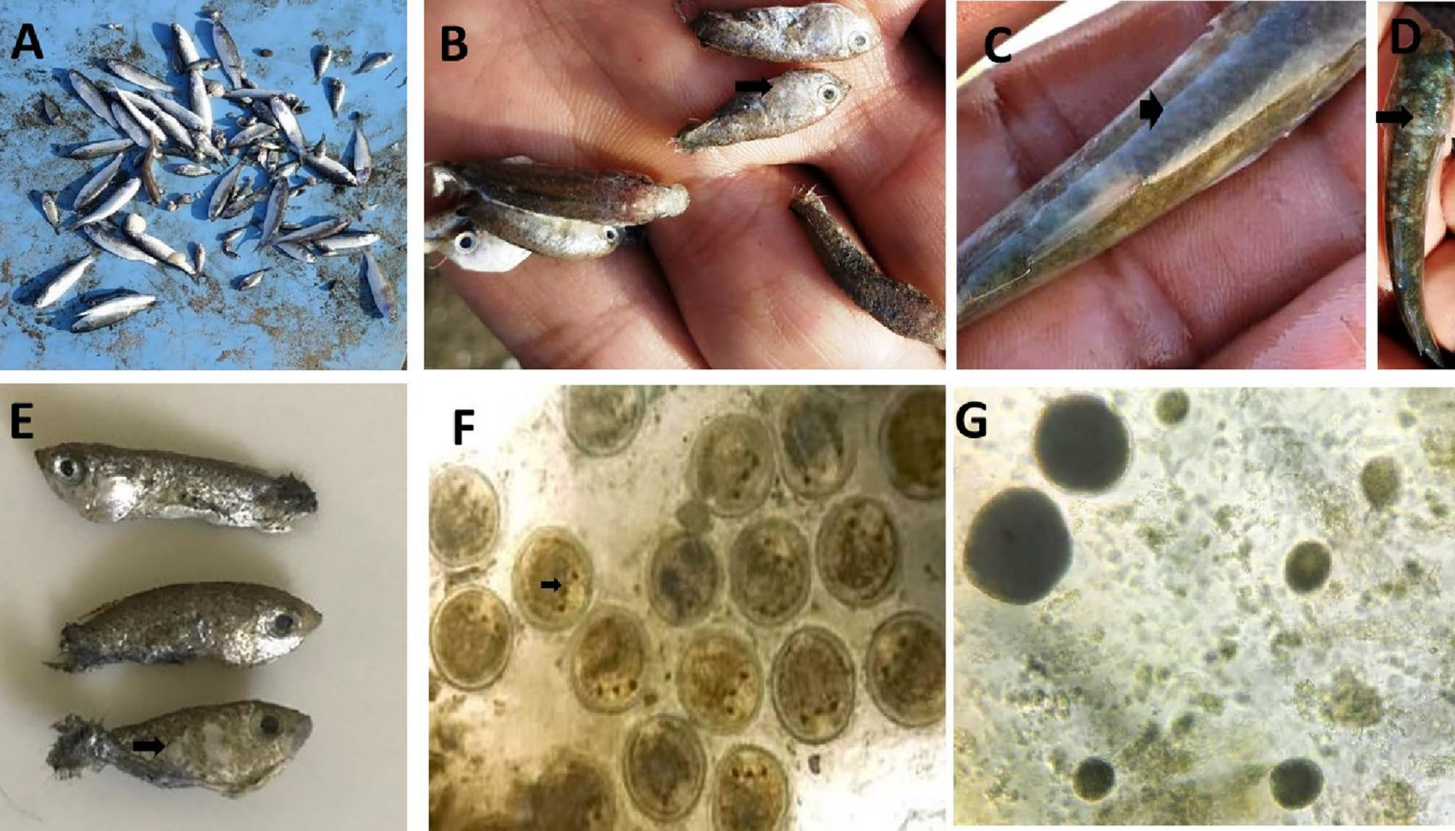
Mass mortality in *Liza carinata* fish with concurrent infection with *Cryptocaryon irritans* and *Amyloodinium ocellatum* infection in marine fish farm. (**A**) Mass mortalities in farmed *Liza carinata*; (**B**) white spots as pinhead-size on skin; fins and gills of small fish due to *C. irritans*; (**C**–**E**) sloughing of the skin of small fish with velvet formation due to *Amyloodinium ocellatum*; (**F**) *C. irritans* with the stage present on the scraping smears was the trophont, note the cytoplasm appeared opaque in color with 3–4 macronucleus, (**G**) *Amyloodinium ocellatum* small to large in size rounded to pear in shape with opaque and dark in color cytoplasm.

The fish showed typical symptoms of respiratory distress.Flashing, anorexia, sluggish movement, and fast opercular movement were all frequent. Fish aggregation at water surfaces and accumulation near water inlets were both common. *Liza carinata* fingerlings were seen close to the margins of the ponds with a lake of escape reflex. Excess mucus was seen in the gills of moribund fish. The skin of succumbed fish was hazy and velvety (Fig. 1), with white spots all over the body and around the eyes (Fig. 1). Some fish succumbed with no obvious gross lesions.

### Prevalence of protozoan infections

The majority of examined fish (90%) were found infected. The greater part (66.42%) of fish examined had a mixed infection, while (23.57%) had a single infestation, either *A. ocellatum* (10.71%) or *C. irritans* (12.85%). The mean *A. ocellatum* intensity in fish tissues was (16.5 ± 2.03) in the skin and (13.18 ± 1.90) in the gills of infected fish. On the other hand, the mean intensity of *C. irritans* was (4.75. ± 1.05) in gills and (7.43 ± 1.45) in the skin of infected fish (Table 1).

**Table 1:** Occurrence of protozoan infections in naturally infected *Liza carinata*.

	No. examined fish	No. infected fish	Prevalence (%)	Parasitic intensity	
				Skin	Gills
*Amyloodinium ocellatum*	140	15	10.71	16.5 ± 2.03	13.18 ± 1.90
*Cryptocaryon irritans*		18	12.85	7.43 ± 1.45	4.75 ± 1.05
*Amyloodinium ocellatum* + *Cryptocaryon irritans*		93	66.42	11.81 ± 3.25	8.34 ± 2.49

Prevalence % was calculated to according to the total number of examined fish. Examined fish were recorded as positive when 1 parasitic trophont was detected. The degree of protozoal intensity was done using protozoal count per examination field.

### Morphological identification

*Amyloodinium ocellatum* trophonts detected in the mucus scrapings were spherical to oval or pear in shape, ranging in length from 37 to 115 (69.76 ± 26 μm); the cytoplasm was opaque in colour, with rhizoids tentacle-like structure for firmly adhering to the gills (Fig. 1). The *C. irritans* trophont, on the other hand, was 325–475 μm (386 ± 1.5 μm) in length and 50–65 (62 ± 0.4 μm) in width. It was rounded to oval or pear in shape with opaque cytoplasm; small to large with crescent shape macronucleus containing four lobes with lengths of 6–10 μm (8.6 ± 0.42 μm) (Fig. 1).

### Molecular identification

#### Amyloodinium ocellatum

**Figure 2:**
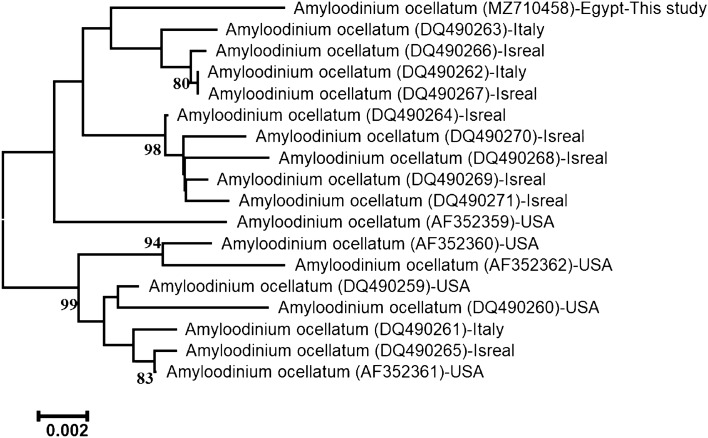
The neighbor-joining phylogenetic tree showed the comparative analysis of ITS rDNA region sequence of *A. ocellatum* infecting *L. Carinata*.

The accession number of ITS rDNA regions of this *Amyloodinium* sp*.* infecting *L. carinata* was MZ710458. The length of the sequenced ITS region was 1318-bp. Depending on its sequence alignment, the present sequence is ascribed to species level to be identified as *A. ocellatum* and firmly embedded within the family Oodiniaceae. The accession number (MZ710458) showed 98.86% identities to that of *A. ocellatum* (DQ490267.1), 98.79% similarity to that of *A. ocellatum* (KU761581.1, KR057921.1, DQ490262.1), 98.71% similarity to that of *A. ocellatum*(DQ490266.1), and 97.58% similarity to that of *A. ocellatum* (DQ490260.1). The neighbor-joining phylogenetic tree of ITS regions of *A. ocellatum* exhibited two major lineages (Fig. 2). The first clade comprises the present *A. ocellatum* grouped with other *A. ocellatum* from Italy and Israel from the Mediterranean Sea.

#### Cryptocaryon irritans

**Figure 3:**
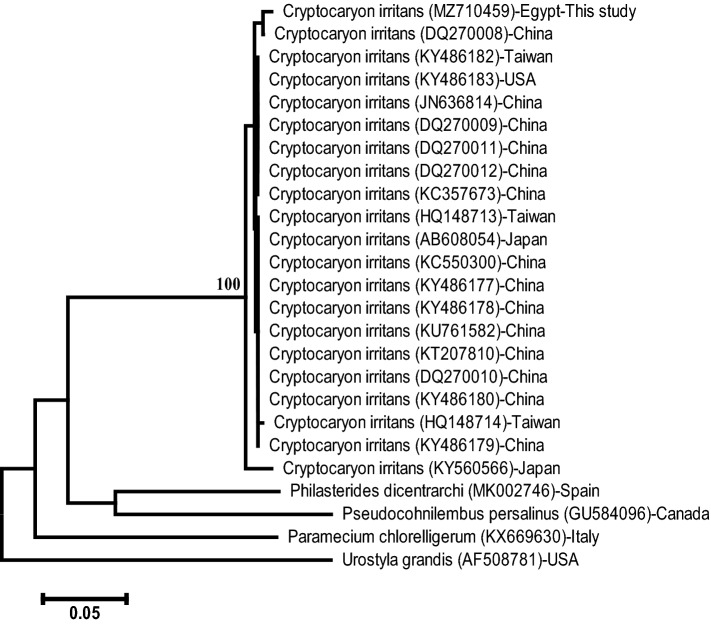
The neighbour-joining phylogenetic tree showed the comparative analysis of ITS rDNA sequence of *C. irritans* infecting *L. carinata*.

The accession number of ITS rDNA regions of this *C.irritans* infecting *L. carinata* MZ710459. The length of the sequenced ITS rDNA regions was 722-bp. Depending on its sequence alignment, the present sequence is ascribed to the species level of *C. irritans* and firmly embedded within the family Cryptocaryonidae. The accession number (MZ710459) showed 99.29% similarity to that of *C. irritans* (DQ270008.1), 99.01% similarity to that of *C. irritans* (DQ270009.1), 98.97% similarity to that of *C. irritans* (KC550300.1), and 98.75% similarity to that of *C. irritans* (KU761582.1, KT207810.1, AF490381.1). The neighbor-joining phylogenetic tree of the ITS rDNA gene of *C. irritans* showed that this sequence is strongly embedded among other *C. irritans* with a 100% bootstrap value (Fig. 3).

### Field treatment trial

During treatment strategery, dead fish should be collected daily and buried in a hygienically based method. At the end of two weeks treatment strategery, the cumulative mortalities were dropped from 65% to about 10% after concurrent daily application of copper sulfate pentahydrate 99% and hydrogen peroxide 40% solutions into the ponds water for one week as well as application of Glutaraldehyde (15%)/Quaternary ammonium compounds 25% (QACs) combination daily for 3 successive days in the ponds water and systemic application of amprolium HCl in the fish feed for 3 successive days.Furthermore, microscopical examination of mucous skin and gills scrapings of random treated fish samples at the end of two weeks of treatment strategery showed a statistically significant decrease in the intensity of both *A. ocellatum* and *C. irritans* (Table 2).

**Table 2:** Treatment trial of mixed protozoal infection in *Liza carinata*.

Treatment trial	Parasites intensity/microscopic field		Cumulative mortality (%)
	Skin	Gills	
Initiation of treatment trial (0 day)	11.81 ± 3.25	8.34 ± 2.49	65
End of treatment trial (14-day)	2.12 ± 0.55	1.05 ± 0.32	10

The degree of protozoan intensity was calculated as previously mentioned in Table 1.

## Discussion

One of the essential requirements for the development of healthy fish is the quality of the farm water. The frequency and severity of parasitic and bacterial infections affecting fish are directly linked to the pond management practices and hygienic conditions of rearing water^33,34^. Poor farm management, such as overstocking, as noticed in the studied earthen ponds, promotes ectoparasites infestations^34^. High fish stocking levels change the balance of environmental and biological factors of the aquatic ecosystem and exacerbate protozoan infections due to higher feed inputs^35^. Fish ectoparasites spread rapidly in crowded aquaculture environments, leading to massive losses^34^.

The physicochemical water quality measures such as temperature, ammonia, DO, pH, and turbidity all significantly impact fish health and disease resistance^36^. The major part of these parameters in the studied farm had exceedingly deteriorated values and was supposed to predispose *L. carinata* fish to *A. ocellatum* and *C. irritans* protozoan infections in agreement with^34^. The inferior water quality measures noticed in the investigated farm could be relevant to poor management practices, including; overfeeding, inadequate replacement of water, and high fish stocking densities in the affected earthen ponds. Furthermore, excessive chicken manure addition and irresponsible fish-pond fertilization exacerbated the problem in agreement with^37^. According to good farm management guidelines, fish should be farmed at an optimal stocking density, and feed rates should not exceed the pond's absorption capacity^38^.

Water temperature (30 °C) affects the growth, establishment, and transmission of parasites'infective stages to new hosts^39^. High water temperature and high salinity levels recorded in the investigated farm favor numerous protozoan infestations with, including *A. ocellatum* and *C. irritants* in agreement with^40^. In addition, the oxygen holding capacity of the water diminishes at extreme high-water temperatures^33^. Furthermore, fish reared at low DO levels, like those in this study, have weaker immune systems and are more vulnerable to illness^33^. Poor management and low DO levels in fish-ponds enhance numerous parasitic infections^33^. The NH_3_ and NO_2_ levels observed were considerably exceeding the suggested optimal limits. High ammonia levels depress the immune system of fish and irritate the gills and skin, making parasitic diseases more likely^33^. High ammonia levels in fish-ponds may be caused by overfeeding and excess feed degradation^33^. Excessive turbidity in farm water may be linked to poor farm management practices such as overfeeding, overstocking, and insufficient water replenishment that increase suspended particles in the farm water^37^. Extreme water turbidity levels in ponds increase parasite infection risk and reduce natural food production^37^.

The results revealed greater phytoplankton biomass of approximately 1675 cells/ml, explaining the farm water's reddish-brown colour. These excessive algal blooms may be relevant to surplus food inputs and the high organic loads in the farm water in agreement with^41^. Algal blooms die-offs induce high toxic ammonia levels and oxygen depletion in farm water; both conditions are detrimental to fish and may lead to infestations. Excessive blooms also cause large pH fluctuations throughout the day, stressing and predisposing farmed fish to succumb to parasitic and bacterial infestations. Fish farmed in such low-quality water, which exactly fits the conditions in the current study, are susceptible to a variety of bacterial and parasite illnesses due to impaired immune mechanisms^34,42^.

Epizooties caused by *A. ocellatum* are well documented in the literature^14,16^. The prevalence of *A. ocellatum* infestations is influenced by various environmental factors, including temperature, and can be recorded all over the year^12^. Multiplication of *A. ocellatum* occurs at a temperature between 16 and 30 °C^43^. Outbreaks commonly occur at higher water temperatures (> 27 °C). The pathogenesis of this parasite is linked to the insertion of rhizoids of trophonts attachment disc into host cells, resulting in degeneration of tissues^14^.

The mean intensity of *A. ocellatum* in *L. carinata* tissues was 16.5 ± 2.03 in the skin and 13.18 ± 1.90 in the gills of infected fish. The findings are consistent with those of Bessat and Fadl^44^, who examined amyloodiniosis in two Egyptian localities, Wadi El-Natroun and El-Max, and recorded average prevalence rates of 84.86% and 39.58%, respectively, as well as average mortality rates of 42.78% and 9.86% respectively. Infections with *A. ocellatum* were intense (> 20 trophonts).

The mean intensity of *C. irritans* in the present study was 4.75 ± 1.05 in gills and 7.43 ± 1.45 in the skin of infected fish. Khalil et al.^45^ recorded *C. irritans* infestations in farmed seabream fish with a higher prevalence of 95.83% in the winter compared to 8.26%, in the summer, respectively. Infestations of *C. irritans* are also common in wild marine fish. Diggles and Lester^46^ studied *C. irritans* in some wild-caught marine fish. Authors recorded the highest prevalence, 100%, in *Acanthopagrus australis* fish with an intensity of 14.6 parasites/fish, while the lowest prevalence, 38%, was recorded in *Gymnocranius audleyi* with 1.9 parasites/ fish. The heaviest infection of *C. irritans* occurred at 17 °C.* C. irritans* multiplies rapidly and penetrates deeply into the integument of its host, impairing the physiological functioning of the skin and gills and therefore increasing the risk of secondary infections^19^. Infected fish showed more excessive mucus production and hyperplasia of epithelial cells in the gill lamellae^18^. The majority of the examined *L. carinata* were infected 90%, with 66.42% having a mixed infection and 23.57% having a single infestation of either *A. ocellatum* 10.71% or *C. irritans* 12.85%. *A. ocellatum* and *C. irritans* with varying frequency have been reported in outbreaks affecting numerous fish species worldwide^12,31,42^.

Morphological characteristics of detected protozoan infestations were identical to *A. ocellatum* and *C. irritans* as described in previous studies^12^. The molecular identification of the present parasites was performed by sequencing ITS rDNA regions flanking 18S and 28S rDNA for *A. ocellatum* and *C. irritans.* The ribosomal internal transcribed spacer (ITS) regions, the small subunit, and large subunit ribosomal DNA genes are well known as important molecular markers for identifying fish protozoans. These genes have been effectively employed to genetically categorize *A. ocellatum* and *C. irritans* in numerous studies^25,26^.

The current scarcity of effective authorized medications and the emergence of antiparasitic drug resistance make it difficult to control fish parasite infections in mariculture fish. Management of parasitic fish infestations requires a thorough understanding of environmental and host factors^32^. Therefore, there is a growing need to develop safe and effective antiparasitic drugs in aquaculture. Copper sulfate, formalin, and potassium permanganates are frequently used to treat various parasitic diseases in fish; however, these chemicals are extremely harmful and cost a lot of money^29,32^. The present findings showed improvement of the treated fish's health after applying the prescribed antiparasitic treatment, as shown by the lower death rate. Mortality dropped to 10% after therapy, compared to 65% before administering the recommended chemotherapeutics, indicating treatment efficiency. The intensity of protozoan infections showed a statistically significant decrease at the end of the treatment trial based on results of microscopical examinations of mucus skin and gills scrapings from treated fish after two weeks of treatment strategery.

Hydrogen peroxide is a promising chemotherapeutic that can control some fungal, bacterial, and ectoparasitic infestations affecting fish^47^. The Center for Veterinary Medicine of the United States Food and Drug Administration approved it to treat some infections in fish. Hydrogen peroxide was successively applied to control some parasitic infestations affecting fish, including sea lice, *Lepeophtheirus salmonis* on fish^47^, protozoan ambiphrya or the trematode *Gyrodactylus* spp. on rainbow trout, and *A. ocellatum* on the Pacific thread fish *Polydactylussex filis*^30^. The most efficient H_2_O_2_ doses for controlling protozoan infestations and monogenetic trematodes affecting fish were 170–280 mg/L administered as a static bath for 30 min. More than 280 mg/L concentrations used for 30-min exposures may be effective in controlling other parasites^48^.

Amprolium is a quaternized pyrimidine derivative that disrupts thiamine metabolism and prevents carbohydrate synthesis by blocking thiamine receptors. Amprolium is one of the safest anticoccidial medicines^32^. The ant-protozoan action of amprolium is based on blocking the thiamine transporter in *Eimeria* sp. meronts accordingly disrupts cell metabolism, inhibits the growth of merozoites and prevents the creation of second-generation meronts. It also slows the formation of sporozoites and affects oocyst sporulation^49^.

Amprolium was evaluated for its anti-protozoan action in aquaculture due to its high safety level and significant efficacy against Eimeria in chicken. In vitro, amprolium chloride was efficient against fish myxosporidium. In addition, amprolium combined with salinomycin effectively controlled *Myxobolus* sp. infection in some marine fish species^50–54^. Eissa et al.^32^ investigated the efficacy of using an amprolium-salinomycin mixture to treat heavy infestations of *Myxobolus episquamali*s affecting earthen pond cultured mullets in a field trial.

## Conclusion

Growing healthy fish necessitates the implementation of favorable water quality measures and appropriate management practices. Poor water quality measures, including extreme temperature, low DO, excessive turbidity, high levels of nitrogenous waste products and dense algal blooms, can predispose fish to numerous protozoan infections. *A. ocellatum* and *C. irritans* are extremely harmful parasites that can harm farmed marine fish causing massive losses. Application of good management practices and efficient control methods are necessary to control protozoan infestations affecting fish. Awareness of aquaculture sustainability and environmental protection are required issues in applying antiparasitic treatments in fish farming facilities. H_2_O_2_ and amprolium have antiparasitic properties and were shown to be beneficial in reducing parasitic infestations of *A. ocellatum* and *C. irritans* protozoa in earthen-pond farmed *L. carinata*.

## Data Availability

All data and materials are available within the article.
